# Dietary and Socio-Demographic Determinants of Serum Persistent Organic Pollutants (POPs) Levels in Pregnant Women in Tehran 

**Published:** 2016-09

**Authors:** Bita Eslami, Batool Hossein-Rashidi, Kazem Naddafi, Noushin Rastkari, Abolghasem Djazayeri, Hossein Malekafzali

**Affiliations:** 1 Vali-e-Asr Reproductive Health Research Center, Tehran University of Medical Sciences, Tehran, Iran; 2 Center for Air Pollution Research (CAPR), Institute for Environmental Research (IER), Tehran University of Medical Sciences, Tehran, Iran; 3 Department of Community Nutrition, School of Nutritional Sciences and Dietetics, Tehran University of Medical Sciences, Tehran, Iran

**Keywords:** Polychlorinated Biphenyls, Polybrominated Diphenyl Ethers, Persistent Organic Pollutants, Pregnancy, Diet

## Abstract

**Objective:** To determine the levels of Polychlorinated biphenyls (PCBs) and Polybrominateddiphenyl ethers (PBDEs) as persistent organic pollutants (POPs) in serum of primiparous women at the third trimester of pregnancy and identify the main determinants of POPs levels such as socio-demographic, lifestyle, and diet in Tehran.

**Materials and methods:** One- hundred eighty five serum samples from two simultaneous case-control studies were collected from September 2013 until August 2015.Ten most abundant PCB congeners (International Union of Pure and Applied Chemistry (IUPAC) congeners 28, 52, 74, 99, 101, 118, 138, 153, 180 and 187) as well as eight PBDE congeners (IUPAC congeners 28, 47, 99, 100, 153, 154, 183 and 209)were analyzed by Gas Chromatography Mass Spectrometry (GC/MS). Multiple linear regression analysis was conducted to explain the relationship between total PCBs and total BPDEs and most detected congeners and some determinants, separately.

**Results:** The mean (SD) age of the participating women was 27.82 ± 5.24 years. The geometric mean (SD) of total PCBs was 2.42 ± 2.26 and total PBDEs was 1.28 ± 1.41 ng/g lipid. Only the PCB 138, PCB 153 and PBDE 153 were detected in 100% of samples. We observed a significant relationship between the time of being indoors and total PBDEs (P = 0.03). Passive smoking was significantly associated with PCB 153 (P = 0.049). The results of the linear regression analysis showed the negative and weak association (P-value < 0.05) between diet (egg and fat and oil consumption) and POPs in this population.

**Conclusion:** It seems the most common route of exposure to PBDEs in our population is indoor pollutants. Meanwhile inhalation of smoke from environment is a route of exposure to PCB 153. Further study is needed to evaluate the effects of socio-demographics and especially dietary intake on POPs level.

## Introduction

Persistent organic pollutants (POPs) comprise a large variety of substance such as Polychlorinated biphenyls (PCBs) and Polybrominateddiphenyl ethers (PBDEs) that are characterized by their ability to persist in the environment, low water and high lipid solubility, slow degradation, and bio-magnification in the food chain (1). PCB have been widely used in heat exchange fluids, in electronic transformers and capacitors and used as an additive in paint, carbonless copy paper, sealants and plastics (2). PBDEs are synthetic chemicals used as flame retardants in a variety of consumer products such as computer circuit boards and casings, television and radios, furniture, textiles, automobiles and construction materials (3). The chemical structure and properties of PBDEs are similar to those of PCBs (4).

Body concentrations of POPs in human biological samples such as serum and breast milk have been analyzed (5-7). Several studies have identified a number of potential health effects associated with exposure to POPs, including cardiovascular effects (8), and neurodevelopment effects (9), type II diabetes (1), endocrine disorders (10, 11), and female reproductive health effects (12-14).

POPs can be transferred through placenta together with breastfeeding, to make up the children’s body burden (15). Therefore, children’s exposure to POPs start from conception and continued in the different life stages and maternal POPs levels in the matrices which have been shown to be the most predictive for evaluation of fetal exposure (16). This is important that these chemicals may have adverse effects on developing fetuses, including intrauterine growth retardations (IUGR), neurocognitive deficits, and hormonal dysfunctions (13, 17, 18).These findings emphasize the sensitivity of early life-stage exposures. Therefore, the evaluation of POPs especially in pregnant women due to exposure of fetus and baby is necessary.

In most of the general population which is not occupationally exposed to POPs, exposure occurs through dietary intake (19). There are many evidences that found the presence of POPs in many types of foods including fish, meat, dairy products, eggs, fruit and vegetables (20-24). Although the results of these investigations were related to dietary habit in each population and the results are different. Meanwhile, maternal POPs levels may be influenced by certain socio-demographic, environmental, or lifestyle factors (25-27).

Different studies have reported varying trends of POPs observed within each trimester of pregnancy (28, 29). The investigation of POPs within a pregnancy should be limited to one trimester for a more accurate evaluation. Meanwhile, some studies established that the measurement of POPs in the first pregnancy of mothers with no history of breastfeeding is more valid than evaluation in multiparous (25, 30).

To the best of our knowledge, exposure of the Iran population to POPs is unknown and no work has been done in this area and evaluation the level of POPs in Iranian population especially in pregnant women is necessary. Therefore, the objectives of the present study was to describe the levels of PCBs and PBDEs in serum samples of primiparous women at the third trimester and identify the main determinants of POPs levels such as socio-demographic, lifestyle, and diet in pregnant women living in Tehran, capital city of Iran.

## Materials and methods

The study population consisted of pregnant women enrolled in three general university hospitals (Arash, Vali-Asr, MirzaKouchak Khan) in Tehran, during a prenatal visit. Appropriate written informed consent was obtained from all participants prior to blood sample collection.

This study was consisted the population of two simultaneously case-control studies which evaluated the association between POPs levels and two complications (Gestational diabetes mellitus; GDM and Pre-eclampsia) in pregnancy that the results are under review in two papers (Data are not published).

All pregnant women were screened between 24-28 weeks of gestation with 75 gr oral glucose tolerance test after an overnight fast which venous blood was sampled. Diagnosis of GDM was based on International Association of Diabetes and Pregnancy Study Groups (IADPSG) criteria. Normal results were a blood glucose < 92 mg/dl at baseline and <180 mg/dl at 1 hour and < 153 mg/dl at 2 hours. A diagnosis of GDM was given if one or more tests had abnormal results (31).

Pre-eclampsia was defined at least two blood pressure measurement that were ≥ 90 mm Hg diastolic or ≥ 140 mm Hg systolic within 14 days of one another after 20 weeks of gestation that accompanied by proteinuria. They had not reported previous abnormal blood pressure. GDM and pre-eclampsia was confirmed by specialist physician.

Normal samples consisted of pregnant women attending the same hospitals for a routine prenatal visit in pregnancy with completely normal test results.

Seventy samples had diagnosed with GDM as the first case group, 70 samples had normal pregnancy (control group) and 45 samples had preeclampsia (second case group). Overall, in total population of pregnant women (n = 185) the main determinants of POPs levels were evaluated in this study.

The inclusion criteria of the study were: (a) to be a primiparous; (b) to be a singleton pregnancy; (c) to be at the third trimester of pregnancy. Meanwhile, participants had not reported any of the following criteria: a previous history of diabetes and pre-eclampsia among first-degree relatives, metabolic and endocrine disorders, renal failure, liver and thyroid disease, chronic autoimmune disease, and anti-phospholipid syndrome and any other previous disease.

Sample collection took place from September 2013 until August 2015. Hospital nurses collected 10 ml blood samples into two 5-mL vacuum blood collection polypropylene tubes from the cubital vein. Within 4 hours the serum samples were separated by centrifugation at 3000g for 15 min and transferred into new test tubes in two parts. A part of serum samples was used to measure the total cholesterol and triglyceride and the remaining parts were stored for measuring POPs. The researcher transferred all collected samples from different hospitals to the Vali-Asr hospital laboratory of the Tehran University of Medical Sciences in iced-packed to avoid thawing and stored at -70º C. A staff member of the Vali-Asr hospital laboratory then analyzed the serum samples for total cholesterol and triglyceride levels using enzymatic kits (Pars Azmoon, Iran).

The trained questioner in each hospital recorded individual information about maternal age, pre-pregnancy weight, height, residual status, gestational age, weight gain during pregnancy, occupation, smoking and alcohol consumption, physical activity and walking by face-to-face interview. Physical activity was defined as having more than 10 minutes of any severe activity per day accompanied by increased heartbeat and breathing.

A 168-item semi-quantitative valid food frequency questionnaire (FFQ) (32), administered by interviewers, was used to assess the usual food and nutrient intake during one year before pregnancy on a daily, weekly, or monthly basis. Portion sizes for each FFQ food item were specified according to the USDA portion sizes and, in some cases, household measures. Food intake was reported in gram per day by the nutrition specialist.

Serum samples were thawed on ice overnight prior to preparation. The analytical procedure details for PBDEs and PCBs are described by Lin et al. 2013 study (33). Briefly, the samples were fortified with Hexachlorobenzene (internal standard) and then mixed with 0.5 ml of pure formic acid and ultrasonicated for 10 min. Serum and formic acid mixture was applied to a combined SPE cartridges comprised of poly divinyl benzene-co-N-vinyl pyrrolidone and Phenomenex Strata-X sorbent (50:50 w/w 200 mg) and filtered. The eluent was cleaned up by a serially combined SPE cartridges, including Sep-pak^®^ Light Silica cartridges and Sep-Pak^®^ plus Florisil which were placed underneath the SPE cartridges, and after SPE conditioning steps the analytes were eluted with dichloromethane under vacuum. The residue was reconstituted in isooctane and analyzed by Gas Chromatography Mass Spectrometry (GC/MS).

PCB and PBDE congeners were analyzed using an Agilant 6890-5973 GC-MS (Agilant Technologies, Palo Alto, CA, USA). Split/ splitless injector set at 250 ºC. GC separation was performed by a HP-5MS column, (30 × 0.25 mm i.d 0.25 μm film thickness). The GC oven temperature was started at 90 ºC and held at that temperature for 1 min. Temperature was then increased to 150 ºC at a rate of 50 ºC/min and held for 1 min. Finally, oven temperature was increased to 330 ºC at a rate of 8 ºC/min and held for 3 min. The flow rate of carrier gas, helium, was set at 1.0 ml/min. Ion source and analyzer temperatures were set at 250 ºC and 150ºC respectively. Transfer line temperature was set at 280 ºC. 

Laboratory assays of POPs were performed at the Faroogh private laboratory. In this study, we focused on the ten most abundant PCB congeners (International Union of Pure and Applied Chemistry (IUPAC) congeners 28, 52, 74, 99, 101, 118, 138, 153, 180 and 187) as well as eight PBDE congeners (IUPAC congeners 28, 47, 99, 100, 153, 154, 183 and 209). PCB and PBDE standard solution that contained our study congeners with more than 95% purity were purchased from Accustandard (New Haven, CT, USA). The limit of detection (LOD) for each PCB congener was 0.05 ng/L and the LOD of each PBDE congeners was 0.1 ng/L. The limit of quantification (LOQ) for each PCB congener was 0.1 ng/L and the LOQ of each PBDE congeners was 0.2 ng/L. The concentration of some congeners in serum samples were less than LOD. We substituted these samples with congener specific LOD divided by the square root of 2 before analysis. Because POPs are highly lipophilic and predominantly carried by blood lipids, in order to obtain lipid-standardized PCB and PBDE concentrations, the lipid content in serum samples were estimated from the total cholesterol and triglycerides concentration using Philips formula (34). Therefore, the serum concentration of PCBs and PBDEs were expressed as nanorgrams per gram of plasma total lipids. 

Statistical analyses were performed using the SPSS software (SPSS, Version 16, SPSS, Inc., IL, USA). The distribution of total PCBs and PBDEs and their congeners were not normal (Kolmogrove - Smirnove test, p-value < 0.05) and right-skewed. Therefore, we tested differences of contaminants levels between the groups by nan-parametric test. A two-sided p-value less than 0.05 was considered significant. Total PCB and total PBDE were calculated by summing concentrations of their congeners. 

All contaminant concentrations were continuous variables for analyses. For linear regression analysis, we transformed contaminants levels to natural logarithm (Ln) to make normal distribution. The Multiple linear regression models were constructed with total PCBs and PBDEs levels as the dependent variables. Meanwhile, linear regression was used for those compounds were detected in 100% of the participants (PCB 138, PCB 153, PBDE 153) by a backward method. In all models the following variables were included as potential predictive variables: age, pre-pregnancy BMI, gestational age, weight gain, being passive smoker, and time of being indoors. Meanwhile, dietary intake of fish & shrimp, meat & products, egg and fats and oils were entered in the models. Variables were selected a priori for inclusion in multiple regression models on the basis univariate analyses (p-value < 0.10) or on the basis of evidence from the literature. All the covariates associated to POPs at a level of p-value less than or equal 0.05 were retained in the model.

## Results

The mean age of the participating women was 27.82 ± 5.24 (median: 27) years. Table 1 shows the total characteristics of study population with respected each group. The arithmetic mean of total PCBs was 3.10 ± 2.26 and total PBDEs was 1.74 ± 1.41 ng/g lipid in total population. 

**Table 1:** Total characteristics of sample population

	**Total**	**GDM**	**Preeclampsia**	**Normal**
	**(n = 185)**	**(n = 70)**	**(n = 45)**	**(n = 70)**
Maternal characteristics				
Age (yrs)	27.82 ± 5.24*	28.76 ± 4.89	28.18 ± 6.27	26.66 ± 4.69
Pre-pregnancy BMI (kg/m2)	25.56 ± 14.26	25.15 ± 4.57	26.23 ± 4.86	22.86 ± 4.17
Gestational age (wks)	34.26 ± 3.38	33.84 ± 3.44	33.58 ± 3.50	35.13 ± 3.09
Triglyceride (mg/dl)	271.02 ± 102.68	279.16 ± 106.79	270 ± 101.14	263.54 ± 100.31
Total Cholesterol (mg/dl)	220.29 ± 43.78	223.04 ± 40.45	212.71 ± 41.53	222.40 ± 48.22
Total serum lipid (mg/dl)	833.37 ± 169.76	830.69 ± 174.44	815.15 ± 161.93	830.69 ± 174.44
Weight gain (kg)	12.74 ± 5.3	11.44 ± 4.61	14.42 ±5.60	12.96 ± 5.48
Education				
University Degree	52 (28.1)	23(32.8)	13(28.8)	16 (22.9)
Diploma	95 (51.4)	38(54.3)	21 (46.7)	36 (51.4)
Under diploma	38 (20.5)	9(12.9)	11 (24.4)	18 (25.7)
Smoking status				
Previous smoker	5(2.7)	1(1.4)	2 (4.4)	2(2.9)
Passive smoker	48 (25.9)	19(27.1)	14 (31.1)	15 (21.4)
Occupation				
Housewife	162 (87.6)	59(84.3)	39 (86.7)	64 (91.4)
Employee	23 (12.4)	11(15.7)	6 (13.3)	6 (8.6)
Use of supplement				
Routine (iron and folic acid)	32 (17.3)	11 (15.7)	11 (24.4)	10 (14.3)
Routine plus calcium	88 (47.6)	33 (47.1)	21 (46.7)	34 (48.6)
Routine plus Multivitamin	117 (63.2)	45 (64.3)	25 (55.6)	47 (67.1)
Routine plus Omega-3	25 (3.5)	10 (14.3)	4 (8.9)	11 (15.7)

**Categorical variables are expressed as number with percentage in parenthesis.

* Continues variables are expressed as mean ± Standard deviation;

**Table 2:** Detection rate and geometric mean of PCBs and PBDEs congeners

**Pollutant congeners**	**Detection Rate** *	**Geometric mean (SD)**
PCB28	121 (65.41)	0.01 (0.02)
PCB52	59 (31.89)	0.01 (0.01)
PCB74	0 (0)	0
PCB99	94 (50.81)	0.01 (0.01)
PCB101	53 (29.73)	0.01 (0.01)
PCB118	162 (87.57)	0.02 (0.02)
PCB138	185 (100)	0.94 (1)
PCB153	185 (100)	1.19 (1.20)
PCB180	146 (78.92)	0.05 (0.14)
PCB187	106 (57.30)	0.02 (0.07)
PBDE28	89 (48.11)	0.02 (0.04)
PBDE47	134 (72.43)	0.04 (0.13)
PBDE99	118 (63.78)	0.02 (0.06)
PBDE100	30 (16.22)	0.01 (0.01)
PBDE153	185 (100)	1.08 (1.28)
PBDE154	17 (9.19)	0.01 (0.005)
PBDE183	86 (46.49)	0.02 (0.03)
PBDE209	39 (21.08)	0.01 (0.01)
Total PCBs	-	2.42 (2.26)
Total PBDEs	-	1.28 (1.41)

* Detection rate presented in number and percentages of samples with levels above the detection limit in parenthesis.

The geometric mean, standard deviation (SD), , and detection rate (value higher than LOD) of each PCB, PBDE congeners and total were manifested in Table 2.

Only the PCB 138, PCB 153 and PBDE 153 were detected in 100% of samples. PCB74 was not detected in any of the samples and therefore no further analysis was conducted.

A strong positive correlation among PCB 153 and 138 (r = 0.78, P-value < 0.001) and PCB 180 and 187 (r = 0.77, P-value < 0.001) was manifested by Spearman correlations. While high correlation among PBDE congeners 209 and 183 with r = 0.70 (P-value < 0.001) was defined. We did not find strong correlation (r > 0.70) across pollutants classes (Data not shown in table).

The relationship between the predictive variables and the levels of total PCBs and total PBDEs is shown in table 3. With respect to maternal characteristics, only the weight gain during pregnancy was associated with the serum levels of total PBDEs, while total PCBs showed no association with this variable. With regards to lifestyle, the level of total PBDEs was slightly associated to being indoors (P-value= 0.07). Meanwhile, women who are passive smoker had higher total PCBs and total PBDEs (P- value = 0.09).

Table 4 shows the results of the linear regression analysis with total PCBs, total PBDEs and 3 congeners that were detected in 100% of samples. We observed a significant relationship between the time of being indoors and total PBDEs (p = 0.03). Passive smoking was significantly associated with PCB 153 (p = 0.049). We found the negative and weak association between egg consumption and total PCBs (P-value = 0.004). Meanwhile the negative association between fats and oils intake with total PBDEs was manifested (β = -0.01, P-value = 0.02).

## Discussion

This study reported the POPs levels detected in serum sample of primiparous women in Tehran, capital city of Iran. In this study, we selected three general hospitals in three regions of city in order to collect samples from all district of city. The evaluation of place of residence showed the sample population in this study consisted most area of Tehran. So we assume this sample population is representative of pregnant women in this city.

**Table 3:** Serum Concentrations (ng/g lipid) of total PCBs and total PBDEs by maternal characteristics and life style factors (n= 185)

	**N**	**Total PCBs**		**Total PBDEs**	
		**Mean ± SD**	**P-value** *	**Mean ± SD**	**P-value** *
Age (yrs)			0.02		0.27
≤ 18	4	4.12 ± 0.99		2.15 ± 1.02	
19-25	53	2.69 ± 2.31		1.72 ± 1.48	
26-35	112	3.11 ± 2.80		1.67 ± 1.40	
> 35	16	4.11± 2.26		2.15 ± 1.37	
Pre-pregnancy BMI (kg/m^2^)			0.86		0.70
< 18.5	15	2.99 ± 2.32		2.25 ± 1.73	
18.5-24.99	97	3.21 ± 2.26		1.67 ± 1.34	
25-35	48	2.96 ± 2.27		1.80 ± 1.53	
> 35	25	2.99 ± 2.33		1.56 ± 1.26	
Weight gain (kg)			0.83		0.02
< 9	50	3.19 ± 2.54		1.47 ± 1.17	
9-12	48	3.25 ± 2.16		1.92 ± 1.46	
12.1- 16	47	3.06 ± 2.52		1.54 ± 1.53	
> 16	40	2.85 ± 1.67		2.07 ± 1.44	
Use electronic equipment					
Computer (min/24h)			0.65		0.14
Never	116	3.23 ± 2.47		1.82 ± 1.41	
1-30	26	2.49 ± 1.49		1.27 ± 0.81	
>30	43	3.11 ± 2.03		1.79 ± 1.65	
TV (hrs)			0.67		0.33
< 2	63	3.28 ± 2.36		1.81 ± 1.44	
2-3	31	3.15 ± 2.15		2.01 ± 1.55	
3.1-5	53	2.95 ± 2.27		1.46 ± 1.19	
> 5	38	2.97 ± 2.23		1.78 ± 1.52	
Being indoors			0.83		0.07
< 22	78	3.02 ± 2.24		1.44 ± 1.15	
22-23	45	3.11 ± 2.38		2.27 ± 1.78	
> 23	62	3.19 ± 2.23		1.72 ± 1.31	
Passive smoking			0.09		0.09
No	137	2.94 ± 2.17		1.62 ± 1.30	
Yes	48	3.56 ± 2.47		2.08 ± 1.67	
Physical activity			0.82		0.60
No	177	3.12 ± 2.30		1.75 ± 1.42	
Yes	8	2.69 ± 0.94		1.44 ± 1.16	

* p value refers to Kruskal Wallis or Mann-Whitney U-test when appropriate; Data are expressed as descriptive value

We found the level of PBDEs is associated with spending more times indoors. Since the PBDEs have been extensively used in indoor applications, indoor air concentrations of PBDEs are generally higher than outdoor levels and are considered as significant pollutants of the indoor environment (35). On the other hand, the present study population consisted mostly of housewives without occupational exposure to POPs due to spending most of their time indoors. As a result, it seems that the most common route of exposure in our population is indoor pollutants such as air and dust.

Schecter et al. evaluated the levels of some PBDE congeners in the US indoor environment, to assess the potential exposure to PBDEs from computer surfaces by computer wipe samples. All samples tested positive for PBDEs (36). However, in the present study, we couldn't find any significant differences between the total levels of POPs in accordance to usage of electrical equipment such as computer or TV.

**Table 4:** Beta linear regression coefficients for total PCBs, total PBDEs,PCB 138, PCB 153, and PBDE 153 after logarithmic transformation (n = 185)

**Pollutants**	**β**	**Standard error (SE)**	**P-value** *
Ln Total PCBs			
Intercept	2.31	0.66	0.001
Gestational age	-0.04	0.02	0.04
Egg	-0.01	0.003	0.004
Ln Total PBDEs			
Intercept	-2.17	1.23	0.08
Being indoors	0.12	0.06	0.03
Fats & Oils	-0.01	0.003	0.02
Ln PCB138			
Intercept	-0.34	0.45	0.46
Age	0.03	0.01	0.05
Egg	-0.01	0.004	0.04
Ln PCB153			
Intercept	1.55	0.72	0.03
Passive Smoking	0.31	0.16	0.05
Egg	-0.01	0.004	0.02
Ln PBDE153			
Intercept	-2.65	1.34	0.05
Being indoors	0.13	0.06	0.03
Fats & Oils	-0.01	0.003	0.03

In all models the following variables were included as potential predictive variables: Age, Pre-pregnancy BMI, Gestational Age, Wight gain, Being indoors, Passive smoking, Fish & shrimp, Meat & products, Egg and fats and oils consumption.

* P-value reported when it was less than or equal to 0.05.

We observed a significant relationship between passive smoking and PCB 153 level. This result was similar with another study concluded that both active and passive maternal smoking increases the neonatal burden with PCBs (37). Higher POPs levels were observed among smoking pregnant women than non-smokers, too (38). Meanwhile, an increased level of PCB 138 with age was manifested in the present study, which in consistent with the results found in pregnant women in other studies (26, 39, 40). 

In contrast to our study finding, previous studies have had different conclusion about the association between dietary intakes of products of animal origin and POPs (20-24). In this study a weak negative association manifested by egg consumption and total PCB and PCB 138 and 153. Not statistically significant association found between fish and shrimp intake and PCB 138, too (β = -0.01, P-value = 0.08). Meanwhile, the reverse association between total PBDEs and PBDE 153 and fats and oils consumption was manifested. 

Various different reasons that could explain the weak association found between diet and POPs levels in the pregnant women who participated in the present study. The first reason maybe depending on when and where it is consumed. Because contamination of the food item in each area is different and the current levels of POPs, could have influenced the dietary intake during adolescence more than habits during one year before pregnancy (25, 26, 40) and the fact the persistent contaminants have been accumulated over a long time prior probably several years and these studied chemicals have long half-life (2-10 year) (41). The last important issue that should be noted is about low per capita consumption of food especially fish, meat, and dairy products in our population in compare with other countries. In this study the mean consumption of fish was 10.84 ± 12.32 gram per day and the mean consumption of meat was 29.77 ± 19.58 gram per day (data not shown in the Table) and if we compare these levels with other countries such as Canada, Finland, Poland and Spain (42), the results shows the low level of consumption in the current population study. Therefore, evaluation of POPs in food samples in different places is needed to accurate estimation of dietary intake of POPs in this country.

This study had some advantages. Firstly, since previous studies reported that breastfeeding is one of the main routes for pollutants excretion (43, 44) and has a significant effect on decreasing the levels of organochlorine pesticides and PCBs in serum (26), the measurement of POPs in primiparous women gives us better estimation of pollutants in human samples (25, 30).

Therefore, we collected samples from primiparous women to eliminate the effect of parity and breastfeeding as confounding factors. Secondly, in order to reduce the effects of various trends of POPs in pregnancy, we collected serum samples from all participants at the third trimester.

We should also considered during pregnancy, total cholesterol will increase on average 58%, especially in the second trimester and triglyceride will increase on average 145% throughout the second and third trimester (45). On the other hand, a physiological increase in plasma volume during pregnancy (46), which could lead to a dilution of pollutants in the plasma of pregnant women. Therefore, higher concentrations of pollutants are expected after delivery and during lactation, which increases the risk of exposure of the newborn through breastfeeding. Future studies should compare the pollutants concentrations during and after pregnancy.

Meanwhile, more survey about the routes of exposure and the trends of POPs, especially in pregnant women due to the effect on their infants and children’s health, is necessary to prevent exposure by taking actions in conjunction with health care providers and stakeholders.
